# A Customized Tool of Incident Reporting for the Detection of Nonconformances at a Single IVF Center: Development, Application, and Efficacy

**DOI:** 10.1155/2021/1126270

**Published:** 2021-10-21

**Authors:** Daria Morini, Jessica Daolio, Alessia Nicoli, Gaetano De Feo, Barbara Valli, Beatrice Melli, Arua Sibahi, Maria Lucrezia Tranquillo, Cecilia Mezzadri, Pietro Ragni, Lorenzo Aguzzoli, Maria Teresa Villani

**Affiliations:** ^1^Center of Reproductive Medicine, Department of Obstetrics and Gynecology, Azienda Unità Sanitaria Locale-IRCCS di Reggio Emilia, Viale Risorgimento 80, 42123 Reggio Emilia, Italy; ^2^Unit of Medical Genetics, Azienda Ospedaliero-Universitaria di Ferrara, Via Fossato di Mortara, 74, 44121 Ferrara, Italy; ^3^Clinical and Experimental Medicine PhD Program, University of Modena and Reggio Emilia, Via Università 4, 41121 Modena, Italy; ^4^Infertility and IVF Unit, University of Bologna, Sant'Orsola University Hospital, Via Massarenti 13, 40138 Bologna, Italy; ^5^Department of Medical and Surgical Sciences, University of Bologna, Via Massarenti 9, 40138 Bologna, Italy; ^6^Clinical Governance Unit, Azienda Unità Sanitaria Locale-IRCCS di Reggio Emilia, Via Amendola 2, 42122 Reggio Emilia, Italy

## Abstract

In IVF centers, risk assessment applies to complex processes potentially accounting for adverse events and reactions that undergo well-established legislative oversight, and nonconformances (NCs), that lack of established tracking systems. NCs account for an integral part of the quality management system, so that their documentation is important. The study evaluated the performance of a customized tool for incident reporting (IR) to track and characterize NCs in a public IVF center. IVF operators used the IVF-customized IR tool to record NCs at the moment of detection or subsequently, and in a time-saving manner during daily practice. From February 2015 to February 2020, 635 NCs were reported leading to the implementation of 10 operative instructions and 3 procedures with corrective strategies. NCs referred to the IVF laboratory were the most numerically meaningful (454/635, 71.5%). The majority (352/454, 77.5%) accounted for NCs related to procedures of sample management; considering the analytical phase as all the procedures involving sample treatment, the intra-analytical phase (176/352, 50%) has always been more subject to NCs compared to pre- (102/352, 29%) and postanalytical (74/352, 21%) phases. Our experience showed that the IVF-customized IR tool is suitable for application in IVF with regard to NC reports and documentation, as it identifies the most vulnerable steps of treatments. It manages NCs over the time, but it requires a contextual understanding of its application in order to avoid NC underestimates that could negatively influence the safety and quality aspects of IVF treatments.

## 1. Introduction

In the field of clinical risk management, the culture of risk and the culture of safety appear to be specular, but they are profoundly different and address distinct issues. The culture of risk allows us to promptly identify errors and their causes, to map the vulnerabilities of a system, to reduce the probability of errors occurring, and to contain damage to patients. The culture of safety aims instead to avoid patients suffering from unnecessary consequences related to care received and that are negative for their well-being [1]. Despite this difference, both cultures are important and should coexist in a system aspiring to patient safety. Moreover, cultures of risk and safety take part in the definition of clinical risk management, established as a set of several tools and actions aimed at identifying, analyzing, evaluating, and processing risks related to healthcare to improve patient safety [2, 3].

Several tools are available to carry out risk analysis proactively and reactively. Proactive tools allow a qualitative or quantitative evaluation of risks with the final purpose of their prevention [4, 5]. In contrast, reactive tools analyze those events that have to be prevented, as there exists a high probability of their recurrence. Both types of tools are applied to many clinical high-risk processes, but their adoption requires a contextual understanding of how the tool contributes to improving patient safety.

Incident reporting (IR) is a reactive tool that is well accepted in safety-critical industries such as aviation as a method for improving safety and is well established in many healthcare contexts, ranging from emergency medicine [6] to primary care [7], intensive care [8], anesthesiology [9], and neonatal practice [10]. This tool provides the opportunity to learn from medical errors and near misses and thereby correct healthcare processes to reduce the risk of recurrence of similar events, thus improving patient safety [11]. Medical errors and near misses involve failures to adhere to accepted standards or protocols, and the corrective process deals with the definition of strategies as well as improvement actions aimed at their prevention. The proper use of an IR system highlights the ability of healthcare systems to work in variable conditions [12, 13], but this is difficult to achieve because it is based on the attitude, awareness of medical errors, and the culture of risk of the personnel involved.

In vitro fertilization (IVF) treatments represent a complex system of clinical, laboratory, and organizational high-risk procedures where it is of crucial importance to protect reproductive cells or patients from damaging events [14–16]. The IVF complexity is due to, on the one hand, the use of high technological laboratory procedures and, on the other hand, the stringent criteria defined by the European Directive 2006/86/EC regarding traceability requirements, notification of serious adverse reactions and events, and certain technical requirements for the coding, processing, preservation, storage, and distribution of human tissues and cells [17–19]. In Italy, the Board of the Region of Emilia Romagna, where our IVF center is located, acknowledged the State-Regions Conference of March 15th/2012 throughout deliberation n.972/2013 that implemented EU Directive 2006/86/EC [20–22].

This legislative achievement constituted a milestone for clinical risk management in IVF, leading IVF centers to tightly satisfy traceability and technical requirements and defining the operative procedures for the notification of adverse events and reactions to IVF treatments. However, deliberation n.972/2013 failed to embrace indications on how to report nonconformances (NCs). Although this could be due to the very low NC rate of IVF laboratories compared with other medical laboratories [12], clause 3.15 of ISO 14001, clause 3.8 of OHSAS 18001, and ISO 9001 : 2000 on quality standards clearly state that the documentation of NCs is an integral part of the quality management system.

In IVF, NCs are defined as any nonfulfilled requirement or deviation from standard protocols and procedures during treatments that can directly or indirectly lead to unplanned consequences, ranging from minimal inconvenience to extreme harm. They are ubiquitous and are not limited to one step of the treatment or sample and patient management. For these reasons, NCs are important enough to be tracked, so that corrective actions can be identified to apply best practices once again to the issue involved. In addition, IVF teams are multidisciplinary, and, therefore, each operator could be accidentally responsible for an “error” that occurs [23, 24]. With this in mind and in the absence of data from the literature on how to report and characterize NCs with regard to the field of IVF, we chose the reactive tool of IR to fulfill the gap of deliberation n.972/2013 on NC management in a more structured fashion. At our center, before the release of the document, NCs were notified through a manual system by laboratory operators only.

Therefore, we developed an IVF-customized tool of IR to identify and record NCs, take the appropriate corrective actions to rectify them, prevent their reoccurrence in the future, strengthen the quality management system, and improve patient safety. Here, we presented the setup and results achieved over a 5-year period. To the best of our knowledge, this paper is the first to describe the management of NCs related to IVF through the adoption of a reactive tool.

## 2. Materials and Methods

### 2.1. Development

The development of the IVF-customized IR tool was coordinated by embryologists from the Center of Reproductive Medicine “P. Bertocchi,” belonging to the Department of Obstetrics and Gynecology at the “Santa Maria Nuova” hospital, AUSL–IRCCS in Reggio Emilia. Following the publication of regional deliberation n.972/2013, a multidisciplinary team was created, recruiting operators with different expertise, such as embryologists, clinicians, nurses, engineers, technicians, and employers, to design and set up an IR tool suitable for use during IVF daily practice. The goal of this tool was to document NCs and follow up any subsequent improvement action. The summary of the organizational activities carried out by the multidisciplinary team and leading to the final creation of the IVF-customized IR tool is given in Table 1.

### 2.2. Structure of Reporting Form

In January 2015, the form of the IVF-customized IR tool (Table 2) was made available via intranet for any member of the IVF center. The form is composed of nine sections with both multiple-choice fields and open text boxes, which allow reporting of all relevant elements regarding the NC captured in a time-saving manner. In the first section, the operator selects the sector in which he/she works: our center is composed of four sectors, including the IVF outpatient service, the IVF laboratory, the surgical compartment of gynecology, and the day hospital service of the gynecology compartment. In the second section, the reporting operator states his/her professional qualification. The date and time of reporting are documented in the third section, whereas in the fourth section, the operator provides a brief description of the NC by filling the open text box.

In the fifth section “NC responsibility,” it is possible to indicate if the NC arose from the IVF laboratory, at the interface between the outpatient services and the IVF laboratory (or vice versa), is secondary to problems not involving IVF operators, or if its origin is due to other circumstances. Specifically, NCs arising from the IVF laboratory involve unwanted events that take place in the laboratory; the responsibility of such NCs concerns the IVF laboratory staff only, i.e., embryologists. NCs secondary to errors at the interface between outpatient service and IVF laboratory include NCs that were made in the outpatient service with an operative relapse in the IVF laboratory (or vice versa), triggering errors; finally, NCs caused by external services, such as pharmaceutical services, supplies, or technician centers, concern unwanted events occurring outside those activities directly depending on the IVF center.

The sixth section “level of NC management” refers to NCs concerning the IVF laboratory only, and it focuses on the level of sample, laboratory, or staff management at which the NC occurred. In the case of NCs belonging to the level of sample management, the reporting operator must choose an option from the seventh section to document when the NC happened, id est during the preanalytical, intra-analytical, or postanalytical phase. Notably, these three phases constitute the whole analytical phase that encompasses all procedures involving the treatment of the sample.

In the eighth section, the operator chooses an option from the list concerning triggering factors potentially responsible for the occurrence of the NC, and in the last section of the form, it is necessary to indicate how the NC was finally addressed.

Of note, reporting NCs has always been voluntary. Both the reporting operator and the operator responsible for the occurrence of the NC remain anonymous. In addition, reporting can be backdated to facilitate and encourage the use of the tool. The tool is based on an electronic database that automatically saves all NC reports. Whenever a form is filled out, an automatic alert system notifies via email the NC reported to the Clinical Risk Manager (CRM) and director of the IVF laboratory and to the director of the IVF center.

### 2.3. NC Extrapolation

Access to the extrapolation system is limited to the CRM only. Extrapolations are made to spreadsheets in which lines correspond to the single NCs, and columns display the information provided by the reporting operator in each sector of the form. The structure of the spreadsheets helps the CRM to develop graphs and statistics to monitor the type, frequency, and content of all NCs registered by the tool. This approach enables the CRM to capture what type of NCs occurs more often and the parts of processes presenting criticisms. The CRM extrapolates and elaborates NCs every six months for proper supervision and once a year. This last process is aimed at discussing and auditing NCs among the CRM, directors, and IVF staff to define strategies, corrective actions, and measures feasible for the final improvement of processes involved. In summary, the system allows NC monitoring and extrapolation at three moments: immediately after NC registration through an automatic alert sent via email as aforementioned, every six months among heads of the center, and once a year among heads of the center and operators for audits.

## 3. Results and Discussion

### 3.1. Results

**Figure 1:**
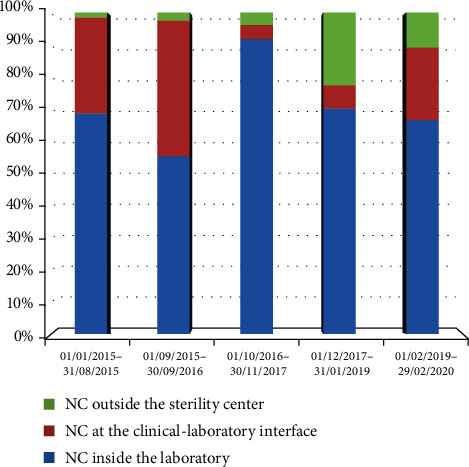
NCs elaborated yearly and grouped by NC responsibility over the study period.

**Figure 2:**
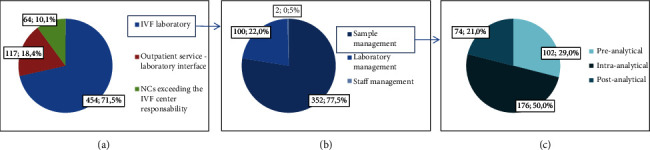
NCs grouped by NC responsibility (a), elaborated by level of management with regard to those ascribed to the IVF laboratory (b). These last were elaborated by sample management phases (c).

From January 1^st^, 2015, to February 29^th^, 2020, a total of 635 NCs were reported, accounting for an average of three daily reports, and twenty operators were involved. NC reports were yearly elaborated and grouped by “NC responsibility” into NCs ascribed to the IVF laboratory, secondary to errors at the interface between outpatient service and IVF laboratory, and ascribed to external services (Figure 1). In the study period, NCs ascribed to the IVF laboratory were the most numerically meaningful, id est 454/635 (71.5%), compared to NCs secondary to errors at the interface between outpatient service and IVF laboratory (117/635; 18.4%), and ascribed to external services (64/635; 10.1%) (Figure 2(a)).

#### 3.1.1. NCs Ascribed to the IVF Laboratory

NCs ascribed to the IVF laboratory were grouped by “level of NC management”: NCs related to the level of sample management were the most numerically meaningful, id est 352/454 (77.5%) compared to those related to the laboratory (100/454; 22%) and staff (2/454; 0.5%) management (Figure 2(b)).

*(1) Level of Sample Management*. During the study period, the extrapolation of NCs referred to the analytical phase revealed that 102/352 (29%), 176/352 (50%), and 74/352 (21%) records were addressed to the preanalytical, intra-analytical, and postanalytical phases of treatment, respectively (Figure 2(c)). Transcription errors in patient records or ID codes on any type of device (dishes, test tubes, labels, goblets, etc.) were the most recurrent NCs reported and related to the intra-analytical phase; other types of NCs, such as deviations from technical operating instructions or protocols approved by the IVF laboratory, wrong localization of dishes into incubator cells, and handling of embryos from patients awaiting results of viral markers not performed in the dedicated laminar flow cabinet, were reported with a lower frequency. Of note, none of these errors had harmful consequences.

**Figure 3:**
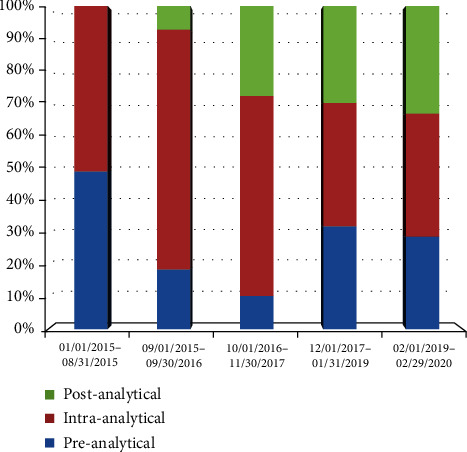
NC frequency related to the phases of sample management over the study period.

Over the first period of its usage, the IVF-customized IR tool revealed that the procedures of pre- and intra-analytical phases were equally vulnerable. The majority of NCs were focused on items regarding the acceptance of incomplete or delayed semen sample reports or the inaccurate preparation of culture media for the next day. We modified the report of semen collection and delivery to the laboratory, trained clinics about instructions to be provided to male patients with regard to semen delivery, and produced an *ex novo* document aimed at supporting embryologists who were preparing culture media and tracking product batches. Starting in October 2016, operators became more aware of these types of NCs, defining a significant drop in their occurrence as confirmed by subsequent follow-ups (Figure 3).

In contrast, starting from the same period, the number of NCs related to the postanalytical phase increased. These last were about recording errors on clinical folders, such as a partial or wrong compilation of data, and the lack of coordination between the IVF laboratory and the outpatient service to store clinical folders at the end of treatment. These NCs showed an increasing trend of detection, ranging from zero in the first two extrapolations to one-third of the total NCs extrapolated in the last period (Figure 3). The corrective action constituted a documented final check of clinical folders carried out and signed by a trained operator and was aimed at verifying the completeness and storage of each folder.

Overall, the corrective actions introduced at this level concerned the adoption of strategies that, taken individually, seem to trend toward triteness. However, if one considers them in the complexity of daily clinical practice, all the corrective actions led to the solving of criticisms that were responsible for operative and time variations in favor of better care during sample treatments.

*(2) Level of Laboratory Management*. Many NCs ascribed to laboratory management were found. The majority referred to failures regarding daily and monthly logbook activities, equipment and environment checks, and missed cleaning of incubators every quarter, nitrogen refill for storage banks, or delayed substitution of incubator filters. The analysis of triggering factors highlighted that these NCs were often due to a forgetfulness factor. This has never compromised the IVF activities.

*(3) Level of Staff Management*. We found NCs relating to failure to update operators' clinical competence, organization charts, and function charts, and all those documents certifying operators' skill training or maintenance at the IVF center. During the study period, the level of staff management was always affected by a minimum number of NCs. Corrective actions have always been applied after audits to update data, limiting the risk of minimal inconvenience.

#### 3.1.2. NCs Secondary to Errors at the Interface between Outpatient Service and IVF Laboratory

During the study period, a variable number of NCs secondary to errors at the interface between the outpatient service and IVF laboratory was detected. This type of NC was ascribed to deviations or events generated after the adoption of modifications to standard protocols, independent of whether they were introduced by the IVF laboratory or the outpatient service.

#### 3.1.3. NCs Referred to External Services

NCs referred to external services represented only a small fraction, id est 64/635 (10.1%), of all the NCs reported. They were primarily involved with the efficiency of external services, including technical, pharmaceutical, and delivery supplies. Starting in December 2017, the role played by external services in our daily practice continued to increase, which is why the related NC detection had to be monitored punctually.

#### 3.1.4. Strategies Implemented

During the study period, a total of 10 operative instructions and 3 procedures were modified, and reactive actions were implemented in light of the results concerning all NC extrapolations. These last results were discussed and audited, and reactive actions were approved among the members of the IVF center. We observed that most strategies were discussed and shared by the team, and most of the strategies had a corrective effect on the issues involved. The efficacy of the strategies implemented was demonstrated by the absence of reports documented through the tool regarding the NC to be rectified. Examples are given in Table 3.

### 3.2. Discussion

#### 3.2.1. Contribution of the IVF-Customized IR Tool to Monitoring NCs in the IVF Laboratory

This paper presents the development, application, and results of a customized form of IR to report and document NCs during IVF treatments. The aim was to monitor adherence to the center's operating procedures and, thereby, improve the quality and safety aspects of treatments in daily practice. The results are discussed over a 5-year period of usage.

The IVF-customized IR tool collected NCs that were notified by different reporting sectors, according to the setting of our specific center. In this paper, we focused on 635 NCs documented by the IVF laboratory: it constitutes the core of every IVF center that can be impacted by activities carried out by different professional figures, is responsible for all the processes surrounding the IVF treatment itself, and accounts for many indicators of quality assurance and control. The ability to disclose NCs decreases the malpractice risk if one admits that NCs occur and is not reluctant to track or react to them [25]. In the study period, the IVF-customized IR tool revealed that embryologists reached a high level of awareness in documenting NCs, as disclosed by the fact that 71.5% of NCs documented by the IVF laboratory were ascribed to the laboratory itself. Notably, these NCs were of minimal inconvenience, and none led to adverse effects for either patients or cells.

The results showed that processes related to sample management were the most vulnerable. We observed that the intra-analytical phase, which constitutes the most delicate phase of IVF processes during which operators handle reproductive cells, has always been more subjected to NCs than the pre- and postanalytical phases. The trend of NCs related to the postanalytical phase is likely justified by a plausible underestimation of these types of events over the first period of tool application. In the following period, the use of the tool fulfilled this gap, enhanced NC perception, and supported IVF staff in acknowledging those events that were previously overlooked. NCs related to the postanalytical phase have probably always been there, but embryologists have become mindful of the importance of capturing them after having experienced the efficacy of the IVF-customized IR tool concerning the identification of actions to improve.

Interestingly, one could speculate that several types of errors persisted throughout the study period. To this matter, the practicality of use of the tool permits us to report NCs constantly. Over the study period, the persisting element was the attitude of reporting, rather than the type of error. This issue varied over the study period, as when audited NCs were rectified with corrective actions, different novel NCs were documented. From a different point of view, the tool performs at such an excellent level in capturing NCs that it seems to have revealed a system afflicted by persistent errors, but this is not true. In addition, the tool offers two operative perspectives: one linear, as it is constantly active over time and never stops tracking NCs, and one circular, as every single NC undergoes a sequence of events that makes it return to the starting point, id est the standard practice, after rectification.

The features of anonymity and backdating contributed to achieving the performance result of our IVF-customized IR tool because they enabled operators to report NCs rather than hesitate, thus fighting against the culture of nonreporting. The NC form is available in the intranet portal of our hospital and can be applied in a time-saving manner. The form requires the compilation of sections in which the operator provides features of the captured NC without revealing either the reporter identity or that of the operator involved. Anonymity is a key element of the tool, as it highlights the fact that the scope of NC notification is to increase the sense of being vulnerable rather than that of guilt at having done something wrong. In addition, the possibility of backdating encourages NC reporting in cases where NC resolution is not immediate by resuming the specific report and integrating the NC resolution into the appropriate section. Taken together, the practicality of use for prompt reporting, anonymity, and backdating allowed us to truthfully photograph what happens in clinical practice without underestimating or overlooking latent errors in gray areas. This was confirmed by the type and frequency of NCs that have been reported over the years and regarding errors in different phases of processes that had been previously ignored.

In our opinion, the performance of the IVF-customized IR tool should also be ascribed to the following reason. The tool can be used to evaluate how actions undertaken to solve NC-related issues improve clinical practice in terms of reducing the recurrence of similar events and, thereby, increasing adherence to approved protocols. The strategy to document NCs combined with the follow-up of corrective changes through the use of a single tool yielded a deviation range from standard practices in the laboratory and, at the same time, allowed us to establish the efficiency of improvements by examining the frequency of a specific NC after the improvement action. Data from the IVF-customized IR tool are available for consultation by the CRM and heads of the IVF laboratory or center whenever needed. The interaction between these figures and embryologists is constant to keep the team mindful of the importance of NC tracking and uniform training methods, independent of the level of the embryologists' experience. The restitutive results regarding both NC extrapolations and improvement actions, either carried out by a small working group or involving all the operators of the IVF center, are precious and highly constructive moments that aim to provide more transparent communication in the final interest of better patient care. From this perspective, our laboratory adopts both the IVF-customized IR tool and other proactive tools of risk analysis to maintain the system under control as much as possible. In this way, the risk of an error occurring is better perceived, acknowledged, and managed, and this makes operators more aware that “the only real mistake is that from which we learn nothing” (John Powell).

#### 3.2.2. Lessons Learned from the Adoption of the IVF-Customized IR Tool as Part of the Quality Management System

The concept of patient safety is an integral part of the healthcare process in which the patient is involved. Quality improvement takes into deep consideration all the issues concerning patient safety and the related actions that are useful in preventing and managing errors, both framed in the context of clinical risk management. If, on the one hand, it is relatively easy to identify the active error, defined as an error taking place between a person and an aspect of a larger system at the point of contact (for example, operating on the wrong eye), then on the other hand, it is more difficult to capture all those latent errors, defined as errors that may go unnoticed for a long time with no adverse effect, but that could be preventable in the majority of cases. In the context of IVF, the concept of patient safety has to be extended to processes regarding human gametes and embryos, with active errors involving adverse events and reactions and latent errors depicting NCs. Italian legislation on IVF formally establishes a system of reporting adverse events and reactions, but it lacks a consistent solution for reporting NCs [20]. This failure fits with the fact that IVF laboratories have a very low NC rate compared with reported NCs in other medical laboratories, especially when one considers the high complexity of procedures performed [12, 16]. Although NCs may not lead to adverse outcomes, they play a role in defining the final quality and safety of care provided to such an extent that they require a robust system of documenting, tracking, and reporting through an appropriate tool. In light of this, the tool that we developed was tailored for IVF to broaden our strategy to monitor errors.

Overall, laboratory medicine procedures are exposed to the risk of adverse events; this risk ranges from 2.7% to 12%, with the majority of events identified in the preanalytical and postanalytical phases [26]. In contrast, the IVF-customized IR tool highlighted the fact that the IVF intra-analytical phase is more NC prone than the pre- and postanalytical phases. This means that medical processes are different, and one must consider their own features to discover vulnerable steps and identify proper risk analysis tools. Although all IVF laboratories and clinics encounter unwanted events, a large taboo still surrounds this issue which is supported by little published data. There is not enough openness about the discussion of unwanted events, thus limiting our ability to learn from each other's mistakes. In fact, the use of tools for risk analysis in IVF is poorly documented and focuses on proactive tool applications whose versatility is well acknowledged [5, 13, 27, 28]. To the best of our knowledge, this study showed for the first time the feasibility of a reactive tool for risk analysis of IVF processes, as it uncovers those nonadverse events that may potentially account for the final safety of care given. In addition, we provided a form of IR with a template that can be adopted by any type of medical laboratory and other entities, and where sections of the form can be modified in accordance with the degree of procedure complexity to be monitored.

## 4. Conclusions

In the context of the quality management system, the main effect of NC reporting through the tool we developed was its contribution to an increased culture as well as awareness of risk among all IVF operators at our center. In this sense, the use of the IVF-customized IR tool contributed to the quality improvement program. Given that, when we act differently and cause an unwanted event, the following reaction can make a constructive difference in avoiding unnecessary and negative experiences or overtreatments in the interest of the best care we can provide. The attitude of reacting is a professional and ethical value that represents the maximum expression of improvement and innovation. Improving patient safety must be a priority of every healthcare system. This becomes achievable if there is a widespread culture of safety among all healthcare providers and when the values of transparency, collaboration between operators, communication, commitment to continuous quality improvement, and the willingness to question their beliefs and actions are shared.

## Figures and Tables

**Table 1:** Steps of development of the IVF-customized IR tool.

Date	Activities
July 2013	(1) Publication of the regional document 972/2013 that introduced mandatory operating instructions in healthcare facilities according to the Italian State-Regions Conference of March 15th/2012
August 2013-August 2014	(1) Definition of NCs potentially occurring in the IVF laboratory (2) Attempts to report and discuss NCs through paper archives (3) Recruitment of an expert and multidisciplinary team (4) Identification of needs to be addressed by the tool (5) Definition of strategy to develop the IVF-customized IR system
September 2014-October 2014	(1) Creation of a form accessible to all users in a time-saving manner (2) Attempts in NCs recording (3) Set-up of record extrapolation to spreadsheets
November 2014-December 2014	(1) Validation of the IVF-customized IR system (2) Availability of the tool inside the intranet of the hospital
January 2015-at present	(1) NCs reporting by all the IVF staff (2) Monthly and yearly extrapolation of records (3) Discussion among IVF staff members (4) Monitoring of corrective action implemented by Clinical Risk Manager

IVF = in vitro fertilization; NCs = nonconformances.

**Table 2:** NC reporting form. NC forms are fulfilled by the reporting operator in each section.

Section 1-reporting sector
(i) Outpatient service (ii) IVF laboratory (iii) Surgery compartment (iv) Gynecological compartment (v) Other
Section 2-reporter qualification
(i) Gynecologist (ii) Biologist (iii) Nurse (iv) Supporting staff (v) Data manager (vi) Collaborating staff outside the IVF center (vii) Other
Section 3-date and time
____/____/____ 00:00
Section 4-NC description (provide a brief summary of what happened)
*Open text box to be completed*
Section 5-NC responsibility (specify the contest to which the NC is chargeable)
(i) IVF laboratory *(go to section 6)* (ii) Outpatient service-laboratory interface or vice versa *(go to section 8)* (iii) Troubles exceeding IVF center responsibility *(go to section 8)* (iv) Others *(go to section 8)*
Section 6-level of NC management (only for NCs chargeable to IVF laboratory)
(i) Sample management *(go to section 7)* (ii) Laboratory management *(go to section 8)* (iii) Personnel management *(go to section 8)* (iv) Other *(go to section 8)*
Section 7-level of sample management (only if the NC belongs to the level of sample management)
(i) Preanalytical phase (ii) Intra-analytical phase (iii) Postanalytical phase (iv) Other
Section 8-NC triggers
(i) Difficulty following instructions and procedures (ii) Fatigue/stress (iii) Taking a shortcut (iv) Lack of supervision (v) Poor team work (vi) Ambiguous procedure (vii) Other
Section 9-report conclusion (describe how the NC was handled)
*Open text box to be completed*

**Table 3:** Examples of strategies implemented.

Level of sample management	Number of monthly reports	Corrective action	Monitoring period	Number of reports after monthly monitoring
*Preanalytical phase*				
When checking documents to accept semen collection, patients forgot to sign all pages	At least 3	Double-sided printing of documents	6 months	0
*Analytical phase*				
Embryos from OHSS patients were observed on day 2 rather than on day 3 as defined by operative procedures	At least 2	Operator retraining	2 months	0
*Postanalytical phase*				
When performing the final check of biological folders, documents were not in the right order	At least 10	Revision of medical records by a professional nurse	3 months	0
When performing the final check of medical folders, the final report of the IVF cycle is missing	At least 5	Revision of medical records by a professional nurse	3 months	0

## Data Availability

Data are available upon request.
